# A supervised machine learning model to select a cost-effective directional drilling tool

**DOI:** 10.1038/s41598-024-76910-z

**Published:** 2024-11-04

**Authors:** Muhammad Nour, Said K. Elsayed, Omar Mahmoud

**Affiliations:** 1 Department of Petroleum Engineering, Faculty of Petroleum and Mining Engineering, Suez University, Suez, 11252 Egypt; 2 Department of Petroleum Engineering, Faculty of Engineering and Technology, Future University in Egypt (FUE), Cairo, 11835 Egypt

**Keywords:** Directional drilling, Digital drilling, Machine learning, Mud motor, RSS, XGBoost regression, Mechanical engineering, Crude oil, Natural gas, Petrol

## Abstract

With the increased directional drilling activities in the oil and gas industry, combined with the digital revolution amongst all industry aspects, the need became high to optimize all planning and operational drilling activities. One important step in planning a directional well is to select a directional tool that can deliver the well in a cost-effective manner. Rotary steerable systems (RSS) and positive displacement mud motors (PDM) are the two widely used tools, each with distinct advantages: RSS excels in hole cleaning, sticking avoidance and hole quality in general, while PDM offers versatility and lower operating costs. This paper presents a series of machine learning (ML) models to automate the selection of the optimal directional tool based on offset well data. By processing lithology, directional, drilling performance, tripping and casing running data, the model predicts section time and cost for upcoming wells. Historical data from offset wells were split into training and testing sets and different ML algorithms were tested to choose the most accurate one. The XGBoost algorithm provided the most accurate predictions during testing, outperforming other algorithms. The beauty of the model is that it successfully accounted for variations in formation thicknesses and drilling environment and adjusts tool recommendations accordingly. Results show that no universal rule favors either RSS or PDM; rather, tool selection is highly dependent on well-specific factors. This data-driven approach reduces human bias, enhances decision-making, and could significantly lower field development costs, particularly in aggressive drilling campaigns.

## Introduction

As technology advances and options increase, the selection process becomes more difficult and time-consuming, drilling is not an exception. Directional drilling is the norm of the industry. Most wells drilled around the globe are directional, thus directional tools never stopped emerging in the market. The most widely used technologies for drilling deviated wells are positive displacement mud motors (PDM) and rotary steerable systems (RSS). Operators differ in which technology do they prefer because each has its advantages. Generally speaking, RSS technology is continuing to occupy the market due to various reasons that will be discussed later on.

The challenge for operators lies in determining which tool will provide the optimal performance for specific well conditions. Historically, this decision has been based on expert judgment and past experience, which can introduce biases and inconsistencies. As the drilling industry evolves, massive data is generated which should be employed for a better decision-making process^1^. With the rise of digital drilling, this data now offers an opportunity to improve the decision-making process through machine learning (ML) techniques.

This paper addresses the need for a data-driven approach to directional tool selection by developing a machine learning model capable of predicting the most suitable tool—RSS or PDM—for any given wellbore section. The novelty of this study lies in its ability to predict and compare total section time and cost for both PDM and RSS by analyzing historical well data. This model not only eliminates subjective bias but also adapts to specific well conditions, offering tailored recommendations for cost-effective drilling.

*Research objectives:*

1. To qualitatively define the favorable conditions for using PDM versus RSS, based on a comprehensive literature review and field experience.
2. To develop and validate a machine learning model that predicts section time and cost for directional drilling using PDM and RSS.
3. To implement a data-driven framework that guides the selection of the optimal directional drilling tool, improving both operational efficiency and cost savings.

## Literature review

Since the introduction of RSS, drilling professionals and researchers have been comparing the effectiveness of PDM and RSS in different environments. The operation principle of PDM and RSS differs in that, PDM principle is to create side-force through bit offset or tilt by incorporating a bent housing and allow the well to change trajectory where the drill string does not rotate. On the other hand, RSS has two variations: Push-the-bit and point-the-bit. Push-the-bit type is centered around producing bending force on bottom hole assembly (BHA) by activating of three pads (ribs), this force is almost proportional to the extent of produced curvature, where, point-the-bit type RSS concept is initiating a bit deflection through using a pivot or a cam to move a steering shaft eccentrically inside a housing called the steering clutch^2^.

The evolution of PDM & RSS has shown continuous improvement that allowed us to drill record-breaking extended-reach wells using both technologies. For example, high-performance PDM (HP-PDM) have been in the market since ’90s and are characterized by a uniform thickness of elastomer rubber which enable it to give higher torque to the bit, thus it can handle more aggressive bits and yield better rate of penetration (ROP)^3^. Stator sections in conventional PDMs deform proportionally to the applied torque from the bit, thus its mechanical efficiency gets lower, while HP-PDM can deliver consistent rotary speeds under high torque conditions^4^.

The mechanical nature of PDM bent housing gives it an advantage over RSS in terms of maximum possible dog leg severity (DLS). So, for the same material and dimensions and thus the same bending strength, maximum DLS achieved by PDM is usually higher. Although newer versions of RSS can achieve DLS comparable to that of PDM but at higher prices even when compared to relatively older RSS. RSS costs more than PDM whoever the service provider is. This can be attributed to better operational performance, relative recency, more expensive electronic components – compared to pure mechanical components of PDM – and naturally the know-how price.

While there are well-defined scenarios where the high cost of RSS makes it impractical^5,6^, and others where RSS is clearly the superior choice^7^—such as in complex trajectories with high rig operating costs—the majority of cases fall into a gray area where determining the optimal tool is less straightforward. Rather than viewing directional drilling tools in isolation, they must be considered as integral components of the entire drilling system, influencing overall performance^8^. In the following sections, we delve into the key qualitative criteria that guide the selection between these two technologies.

### Main selection criteria

#### Equipment selection

Using PDM or RSS has a dominant effect on drilling operations from the very beginning. PDM is more versatile when rig selection is an issue. Because it depends on downhole hydraulic power of mud to rotate the bit, a situation where a weak top drive system (TDS) or rotary table is in service can be handled, this may not be the case for RSS which depends entirely on rotary speed delivered from the surface.

PDM add more burden to the mud pumps as it has its intrinsic differential pressure, this would be a factor while choosing liner size for mud pumps and if not optimized may jeopardize hole cleaning efforts^9^.

RSS (push or point) and PDM require different bit specifications^10,11^. PDM has a wide range of variation in terms of torque/speed combination, this makes the process of bit selection more flexible than that of RSS. Some formations need a specific type of bit to be drilled, which may not be compatible with RSS. These facts may necessitate the use of PDM in some situations^3^.

#### Drilling parameters and directional performance

During the drilling, weight on bit (WOB) has different effects on the building capability of PDM and RSS. While PDM is deployed, higher WOB assists in increasing build-up rate (BUR). This is not the case while using push-the-bit RSS. Applying high WOB while using RSS will not allow enough time for side cutting of the bit and thus reduce achievable DLS. The mechanical setting of PDM, although it gives an advantage in terms of achievable DLS while slide drilling, is a major limiter for rotary speed^3^. The higher the bent housing angle, the lower is the permissible surface rotary speed.

Using RSS, the effect of rotary speed is not the same as the current knowledge of conventional rotary assemblies, increasing rotary speed while using RSS increases the achievable BUR as it improves the side cutting in a similar manner to time-drilling applications.

Regarding drilling performance, bit ROP (instantaneous ROP) achieved using RSS is usually higher than that of PDM while performing directional work (build, drop, or turn). This is mainly due to eliminating sliding intervals, in which PDM usually suffer from hanging problem, although it may be solved to a certain extent using down hole agitators^9,12^.

However, cases were reported where ROP of PDM was 69% higher than that of RSS on average. This may be attributed to increased bit rotary speed in case of PDM^3^.

While being used throughout this paper, average ROP is defined as the total footage drilled per operation hour regardless of if the bit was actually drilling or other operations (e.g., connection, tool face adjustment, downlinking…etc.) interfered. Average ROP by definition is always less than on-bottom ROP. Although RSS is in most cases faster than PDM within intervals of directional work, this may not be the case for hold intervals. PDM has the advantage of extra downhole rotary speed pursued by its design. Rotary speed is one factor that affects ROP positively. So, the contrast of average ROP depends on the ratio of directional work intervals to the total interval length. Besides on-bottom time drilling, there are other operations that consume time:

- Orienting the tool face (PDM)^13^.
- Sending downlinks (RSS).
- Survey measurements, connections, wash, and ream (both).

It should be noted that while sending downlinks to RSS, the directional driller may continue drilling with reduced ROP or stop drilling entirely depending on how soon he needs the tool to respond to the sent orders^14^.

In some times, the overall improvement in ROP as a result of RSS deployment does not match the increased cost^15^ and motorized RSS is considered^16^. Motorized RSS are used to improve drilling performance^17^ and in some reported cased the improvement reached 39%^18^.

#### Hole quality

Hole quality is one of the most important end results required for a successful drilling operation. Hole quality affects how deep a well can be drilled, at what rate and the usable lifetime of drill string components. It also affects casing running, logging and completion operations^19,20^.

Tortuosity which simply describes unwanted deviation from a planned curve or hold Sect^21^ can be measured on macroscopic (between standard survey stations) or microscopic scale (using continues survey measurements or high frequency borehole imaging tools). A uniform curve has a zero tortuosity, where changing build or turn rates will increase it. So, drilling with PDM gives much more tortuosity than drilling with RSS^5,20,22^. Also, macroscopic tortuosity measured for PDM run will be less than actual one, as the survey station could have a slide/rotary transition which induce more hidden tortuosity^23^.

While RSS is capable of achieving uniform DLS without the stair-step profile of PDM, it must be stressed that directional driller – if not competent – can cause the same stair-step phenomenon. Assume that inclination is required to change from 63° to 65° along the next 50 ft from 5000 ft to 5050’, the RSS side-force has to be adjusted for a gradual inclination increase of 4° per 100 ft to reach 65° at 5050 ft. Directional driller must not for example use a higher force, reach 65° at 5035 ft and then hold the angle till 5050 ft, if so, there is no gain from using RSS in terms of hole quality.

During slide drilling with PDM, the hole should be –theoretically – in gauge, but during rotary intervals, the motor drills a slightly over-gauge hole. Although RSS is generally better than PDM in terms of local DLS, operational mode^24^ and human factor still affect the hole quality and resultant tortuosity^23^.

Less tortuosity of hole drilled with RSS compared to that of PDM results in less drag and thus extend the drilling envelope to farther lateral displacements^3,5^. In some cases^25^, friction factor (FF) using RSS was 28.6% lower compared with PDM, indicating better hole condition and less tortuosity. In addition, continues rotation – which is one factor with great effect on hole cleaning – gives an advantage to RSS over PDM as the drill string is continuously rotated while drilling with RSS. As a result of continuous rotation and better hole cleaning, pipe sticking is way less potential while using RSS. Nevertheless, while the probability is less, the consequences are more severe. If RSS is stuck and cannot be fished, the Lost-In-Hole (LIH) cost is much greater due to the price difference between RSS and PDM.

#### Drilling mechanics and dynamics

Vibration is a major concern while drilling directional wells. Types of vibrations can be summarized as:

- Lateral vibration,
- Axial vibration, and.
- Torsional vibration (stick/slip).

Axial vibration is more prominent at shallow depth and can be damaging to surface equipment, this type of vibration can be overcome by using shock absorber above the bit. Lateral vibration is the most damaging type and can easily cause a twisted-off drill string component.

While torsional vibration can damage downhole sensors and drill string components. Torsional vibration can arise as a result of drill string/hole friction or from drill bit/formation interaction. If PDM is used, it acts as a dampener or decoupling device that prevents vibration transition from drill bit up the drill string while rotary drilling^4,26,27^, and no torsional vibration occurs in the first place during slide drilling phases. In area where stick/slip is known to be risky, high sampling rate recording may be essential to avoid missing severe micro stick/slip events^28,29^.

Vibration in general has a mechanical effect on PDM, where in push-the-bit RSS -which is more prone to stick/slip^11^ - it can damage RSS either mechanically (wash-out, fatigue or twist-off) or hydraulically by damaging hydraulic chamber behind the pads.

In some cases, decreasing surface RPM, will aid in alleviating downhole lateral vibration, that why PDM will be better than RSS in these cases^30^.

In addition to recommendations from service companies, some researchers found aligned results^30^. In their work, they suggested not exceeding 5 g root mean square (RMS) lateral vibration level to keep chances of twist-off low. Mitigating downhole vibration allows optimal application of drilling parameters and faster drilling operations^31^.

When discussing stresses on drill string, elaboration is required. One factor that affects stresses is rotary speed. From one perspective, using PDM, reduces the rotary speed due to two factors:

- The bent housing angle dictates a certain rotary speed limit to avoid excessive stress on this weak point.
- Due to downhole rotary speed inherent in PDM design, less surface rotary speed is required to achieve the same drilling performance.

In this regard, PDM causes less stresses than RSS^4^. But RSS gives a smoother hole and thus less friction forces (mainly T&D), in addition, sliding is the main initiator of helical buckling^32^, so RSS reduces the incidence of buckling through elimination of sliding^3^. BHA design also greatly affects the vibration tendency of a certain BHA, whatever directional tool is used^33^.

#### Well design and geologic settings

Generally, RSS is more preferred during drilling of long laterals due to its better weight transfer qualities^34^. PDM is not preferred as well, if critical geosteering is planned due to inherent micro-tortuosity and longer time required to correct well path^35^. Here is a merit of RSS over PDM due to close and continuous control of near bit inclination (NBI) as a result of its proximity to the bit and thus a more reliable well placement^36,37^. On the other hand, RSS (push the bit type) is also not the best option in cases where formation washouts are expected^18^, where formation is soft^38,39^ or has too high or too low UCS^40,41^, although some operators have applied customized solution for this problem^9,42^. Point-the-bit RSS system is better than push-the-bit one at these cases as a result of less sensitivity to hole wash-out and its ability to achieve required DLS^39^.

It must be noted that, although a certain DD tool (either PDM or RSS) would have a certain theoretical DLS capability^43^, practically, the maximum attainable DLS is usually less than the published values^9,44,45^.

#### Special applications

In special applications like directional drilling with casing, RSS may be better in small holes due to less directional capabilities of small size PDM BHA^46^. Also for drilling near the lease boundary, a vertical-seeking RSS would be more preferrable due to its instantaneous correction of the inclination^47^. PDM on the other hand, operates more efficiently in case of open hole side-tracking, although RSS proved to be successful under certain conditions^39,48^.

The most relevant studies concerning the selection criteria between PDM and RSS are summarized in the Table 1, highlighting their key findings and limitations in relation to our research focus. These limitations, particularly in the areas of quantitative analysis and data-driven approaches, form the foundation upon which this research is built.

**Table 1:** Summarized critical literature review for relevant works.

References	Year	Main study focus	Finding/Strength	Limitations
^18^	2024	Directional drilling performance optimization in brown fields.	HP-MRSS BHA gave 29% ROP improvement over standalone RSS.	No direct comparison between RSS and PDM performance.
			HP-PDM yield 17% improvement over standard PDM.	
^24^	2020	Effect of local DLS on stuck pipe incidence and completion running.	Point-the-bit and PDM are more prone to problems arising from local DLS than push-the-bit RSS.	No quantitative comparison between PDM and RSS in terms of effect of casing/completion running or drilling T&D.
^10^	2020	Effect of bit design on steerability and performance.	Drilling performance and directional steerability are improved using both PDM and RSS when shorter PDC design was tested.	No analysis done for the extent of relative improvement between PDM and RSS.
^44^	2022	Optimum selection of DD technology and bit for drilling the required path with minimal BHA-related NPT.	Book values for DD tools capability are seldom met during operations.	No roadmap provided to choose DD technology based on planned well path.
^40^	2008	Effect of formation strength on RSS steerability.	RSS directional performance is best where formation UCS is in a sweet spot between 8 and 20 MPa.	No similar investigation done for PDM.
^45^	2001	PDM performance in terms of power output regarding motor geometry and operating parameters.	A correlation between mechanical power output of PDM relative to hydraulic power input is developed.	The correlation is based on torque output of motor, no mention for drilling performance of motor.
^49^	2018	Focus on directional drilling automation for PDM using machine learning for better economics in terms of ROP, hole quality and less physical presence of people (POB).	Advisory system for automated directional drilling was suggested.	Focused on PDM operations only in terms of sliding and tool face.
				Effect on drilling performance is not clear.
^50^	2017	Analysis of directional drilling data for RSS and PDM runs from 2014–2015 within Saudi Aramco.	For a better performance, directional tool has to be periodically replaced if a trip is done, e.g. bit change trip.	It is just a simple statistical analysis for RSS and PDM runs with no deep insights about directional tool selection criteria.
				No clear data about MTBF for both systems.
^51^	2011	Drilling optimization in a SAGD project via implementing newer technologies.	RSS was better than PDM in terms of well placement and cost-effectiveness.	The study was a case study with specific circumstances that cannot be generalized.
^14^	2020	A deep learning model that detects real-time downlinking (DL) events for RSS.	Compare DL drilling performance against PDM slide drilling.	Neglect all under-the-hood variables and focus only on detecting controlled-drilling periods.

**Fig. 1:**
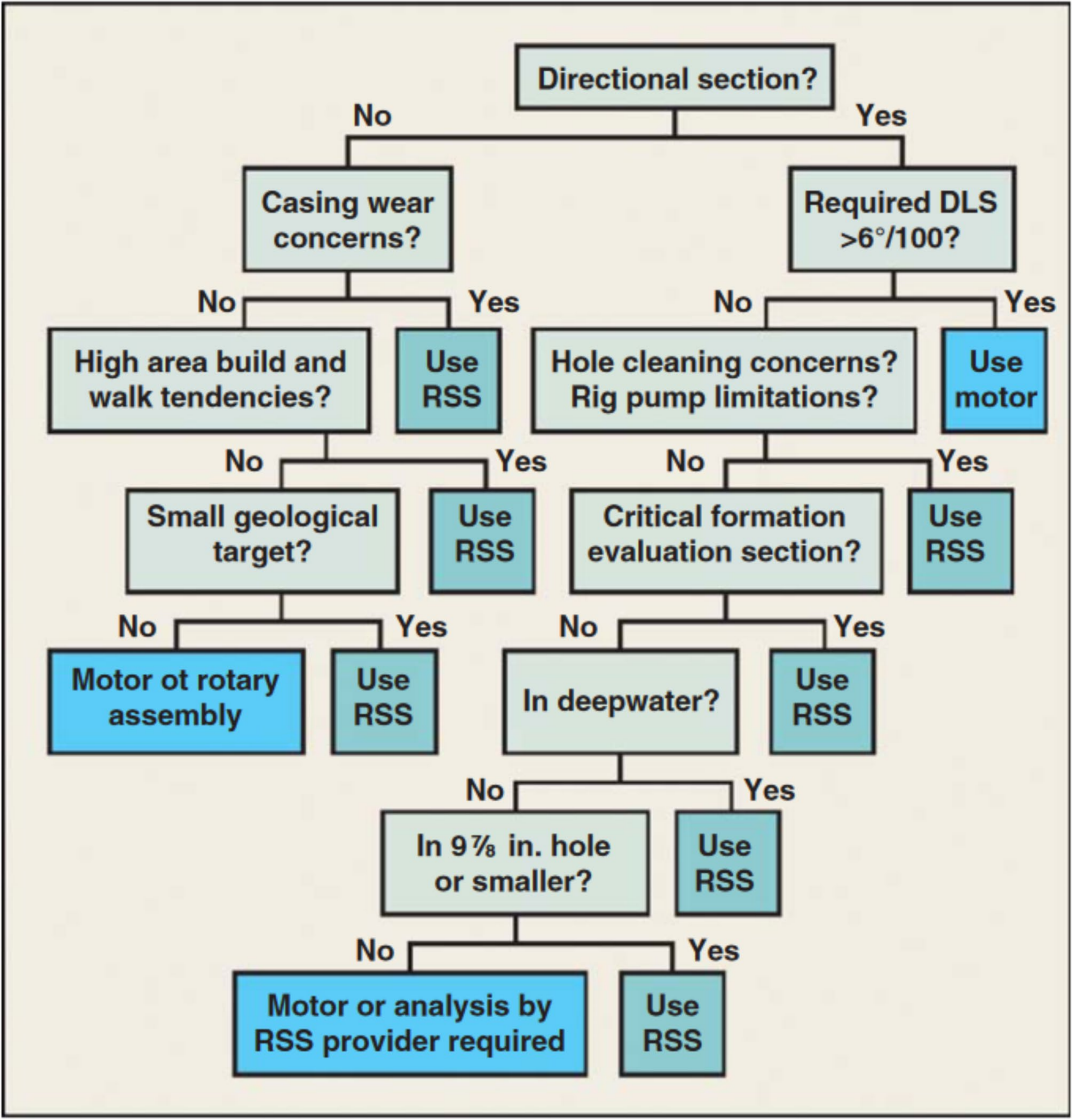
RSS vs. PDM flow-chart guide for best-fit steering selection in GoM^19,52^.

While the body of literature extensively explores the selection of directional drilling tools, most discussions remain qualitative, relying solely on expert judgment and descriptive analyses. Numerous studies outline the advantages and disadvantages of each tool based on case studies, field experiences, and theoretical assessments. The closest approach to a useful framework was a flowchart (Fig. 1) presented in^19^, which was cited from^52^. However, this flowchart remains a qualitative guideline specific to a particular regional context, offering limited generalizability beyond that setting.

There is a critical gap in the literature when it comes to the application of quantitative methods, particularly data-driven and machine learning techniques, to objectively evaluate and predict the optimal tool for specific well conditions.

Despite the growing integration of machine learning in various domains of the E&P^53,54^ and drilling industry^55–57^, no existing research, to the best of the authors’ knowledge, has applied these techniques to systematically predict and compare directional drilling tool performance in terms of cost and time. This research, therefore, represents a pioneering effort in the field, offering a novel, data-driven framework that bridges the gap between qualitative insight and quantitative prediction. By leveraging historical well data and advanced machine learning algorithms, this study transforms the traditionally subjective decision-making process into a scientifically grounded, automated tool-selection model. By addressing these gaps, the study aims to advance the existing body of knowledge and provide a more robust framework for directional tool selection. This groundbreaking approach sets a new precedent in the industry, positioning the research as a first-of-its-kind in the quest for optimized directional drilling operations.

### Machine learning and drilling industry

Drilling operations produce vast amounts of data. Processing these data takes many forms and gives different outcomes. Old techniques like manual offset analysis were very useful in the last decades, but as drilling became more complex, more advanced techniques are required. Here comes the role of machine learning (ML) models. These models can handle a huge amount of data, maintaining computational resources and providing the best results^58^. Two main categories of ML models exist namely supervised models and unsupervised ones. In the first type, data are already labeled, and the model is trained on the training dataset to expect the outcome of validation and test data using several algorithms of regression or classification^59^. Whereas in unsupervised models, datasets are unstructured, and the model objective is to define a certain pattern in the dataset. Applications of ML in drilling are accelerating and most major oil and gas operators are becoming more inclined towards “Digital Drilling” as the drilling performance is correlated to the level of data implementation^60^.

Digitalization is invading oilfield. As an example, fully digital directional drilling with no physical presence of directional drillers on rig site has been adopted by many service providers^61^. Even more, morning reports are on way to full automation^62^, where human input would be minimum.

Many equations were empirically produced due to lack of satisfactory computational power in the past, but with advancements in ML applications, better data processing ways are attainable^63^.

Artificial Intelligence and ML were used in many drilling applications, including real-time well placement for better productivity^64^, pore pressure prediction^65,66^, prediction and classification of lost circulation events^67–69^, drilling dynamics and vibrations^70^, stuck pipe events avoidance and classifications^71^, drilling fluids specifications^72,73^, bit selection and optimization^74–76^, ROP prediction and optimization^63,77–80^, well cost estimation^81^, monitoring of lost time^82^, BHA design^83^ and walk tendency^84^, and well integrity^85^.

The industry is seeking better performance by making best use of digitalization to save rig time and well cost^86,87^, in one case, the efforts caused about 20% reduction in well time^86^. The current paper is aligned with the ultimate industry goal of optimizing drilling time and cost.

With the qualitative factors influencing tool selection thoroughly examined and the implementation of machine learning in the drilling industry well-documented, the foundation is set for developing a quantitative, reliable, and generalizable system. The following section outlines the materials and methods used to construct a data-driven, unbiased framework capable of determining the optimal directional drilling tool for any given subsurface and operational conditions.

## Materials and methods

For a successful directional drilling operation, any operator would decide to run PDM or RSS based on:

- Better drilling performance (less total section time including next casing running), or.
- More economic operations (less section cost), or.
- More preferably both.

### Problem formulation

The time of a certain section is the sum of drilling time (involving other operations like surveys, etc.), tripping time, time to make-up or lay-down BHA. To assess the effect of directional technology on total section time, the time taken to run casing after finishing the section is taken into account as it is - besides tripping time - representative of hole quality. To estimate the section cost, the timings stated above are transferred into cost. And any flat cost of directional tools should be added besides expected value of lost in hole cost (LIH cost multiplied by probability).

To calculate the total section cost and time associated with either RSS or PDM, the following equations are proposed by the authors (Eq. 1 through 3), note that in Eq. 3, the subscripts RO and RC refer to running in open hole and cased hole, where the subscripts PO, PC refer to pulling out of open hole and cased hole, respectively:

1 $$T={T_{MU}}+\frac{F}{{RO{P_{AVG}}}}+{T_t}+{T_{LD}}+\frac{F}{{{S_c}}},$$

2 $$C={C_f}+\left( {{T_{MU}}+\frac{F}{{RO{P_{AVG}}}}+{T_t}+{T_{LD}}} \right)\left( {{C_r}+{C_t}} \right)+~\frac{F}{{{S_c}}}{C_r},$$

3 $${T_t}={N_{RC}}\frac{{TD~ - ~F}}{{{S_{RC}}}}+{N_{RO}}\frac{F}{{{S_{RO}}}}~+~{N_{PC}}\frac{{TD~ - ~F}}{{{S_{PC}}}}~+~{N_{PO}}\frac{F}{{{S_{PO}}}},$$

where C = Total cost ($); T = Total time (hr); T_t_ = Tripping time (hr); C_f_ = Flat charge of either PDM or RSS; T_MU_ = Make Up time of Tool (hr); F = Footage drilled (ft); ROP_AVG_ = Average run ROP including tool face adjusting, downlinks, surveys, connections, wash, and ream time (ft/hr); S_RC_ = Tripping speed while running within cased hole (ft/hr); S_RO_ = Tripping speed while running within open hole (ft/hr); S_PC_ = Tripping speed while pulling within cased hole (ft/hr); S_PO_ = Tripping speed while running within open hole (ft/hr); N = number of trips (with the same assigned subscript); T_LD_ = Time of lay down (hr); C_r_ = Rig operating rate including service companies ($/hr); C_t_ = Tool operating rate (PDM or RSS), including all related BHA components ($/hr); TD = Section total depth (ft); S_c_ = Casing or liner running speed (ft/hr).

To accurately calculate time and cost, certain variables, such as BHA assembly and lay-down time, rig and tool rental rates, tool flat costs, tripping speed within cased hole, section footage, and total depth (TD), can be directly assigned using predetermined values. However, to mitigate uncertainty and avoid introducing bias into the decision-making process, three critical variables—average run ROP, tripping speed in open hole, and casing/liner running speed in open hole—cannot be assumed. To address these uncertainties, we developed a series of three advanced machine learning (ML) models. These models predict bit ROP, section average ROP, and tripping/casing running speeds, effectively integrating a broad range of drilling and operational parameters. This approach provides a rigorous, data-driven foundation for accurately estimating section time and cost for both PDM and RSS, thereby enabling informed, quantitative tool selection.

### Modeling aspects and feature engineering

**Fig. 2:**
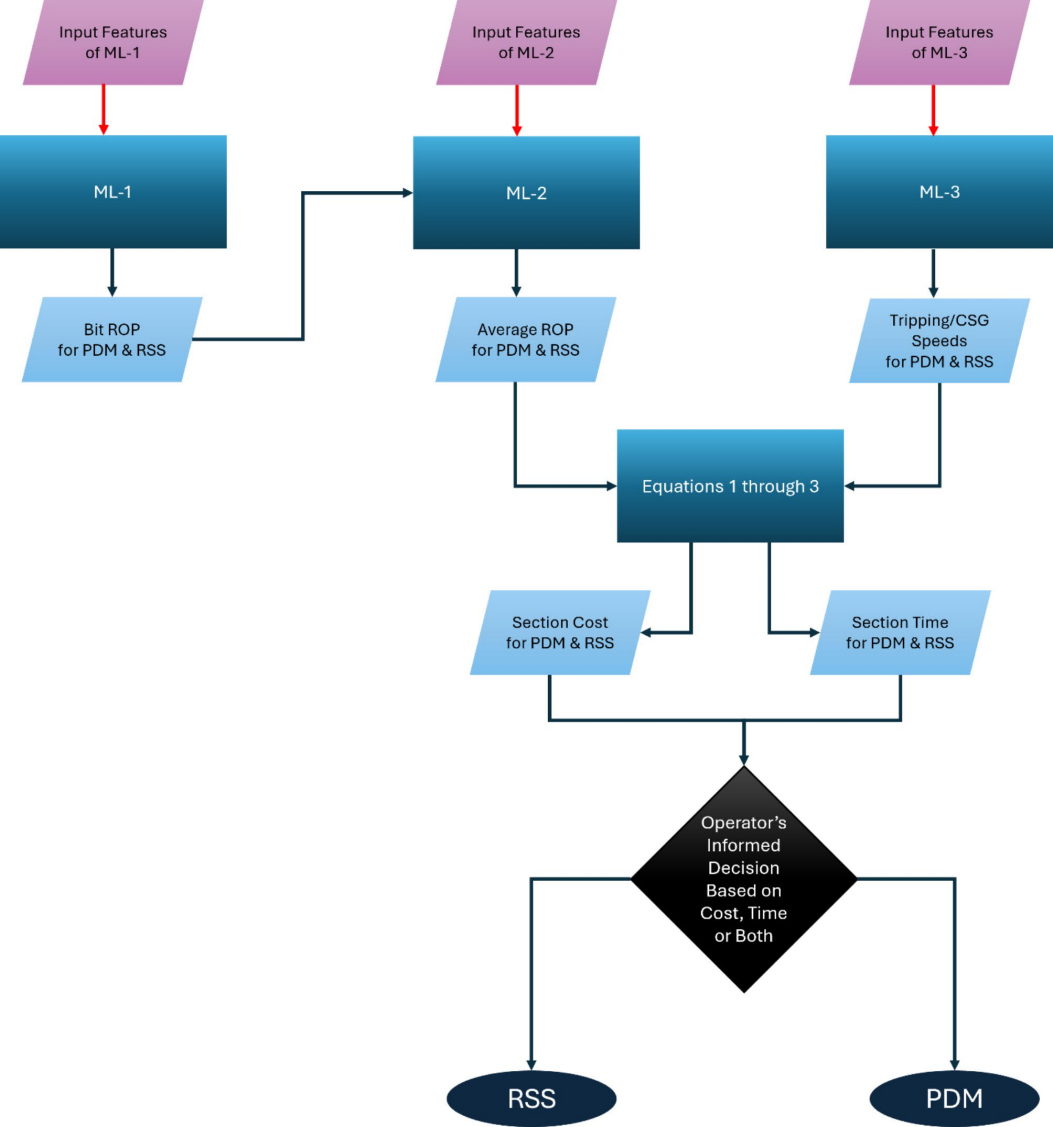
Architecture of the algorithm.

In the next three subsections, an explanation of the aspects for each model is given coupled with criteria of selecting features (feature engineering) for the specific model. Figure 2 represents a comprehensive flow chart for the workflow.

#### Model-1: bit ROP prediction (instantaneous or bottom ROP)

The first model is designed to predict bit ROP, a key determinant of drilling efficiency. It depends on many variables like: directional technology used (either PDM or RSS), formations to be penetrated, which in turn can vary in drillability between fields^88^ and even at different depths within the same field, hole size, mud weight and type, inclination, azimuth^89^ and DLS, drilling parameters (WOB, flow rate and rotary speed), and bit type (tri-cone or PDC) and condition (new or re-run)^90^. A regression-based ML approach was used to map the complex relationships between these input variables and the bit ROP, enabling accurate predictions across different formations and well designs.

#### Model-2: section average ROP prediction

Building on the predicted bit ROP, the second model calculates the section average ROP, a vital input for determining both section cost and time. Average ROP takes into consideration time for tool face adjusting, downlinks, surveys, connections, wash, and ream. Intuitively, average ROP is a fraction of bit ROP, how much is this fraction depends on few variables like:

- Directional technology: PDM and RSS have offline time which does not contribute directly to drilling like survey, wash and ream time, time for adjusting tool face in case of PDM and sending downlink in case of RSS. This offline time may differ between PDM and RSS.
- Rig: a rig equipped with TDS will have greater average ROP than one equipped with Kelly if both drill at the same instantaneous ROP. Also rig Horsepower (HP) and crew performance affect how efficient are the operations.
- Hole size also may affect how fast wash and ream are done.
- Bit type has measurable effect on wash and ream time due to some bit intrinsic features (e.g., back-reaming cutters within PDC bit).
- Inclination and DLS also affect wash and ream time and frequency. Driller needs to be more careful at zones of higher inclination and DLS to avoid hole pack-off during wash and ream.
- Mud type also has a potential effect of wash and ream time. Oil-based muds are known to improve friction profile within the well and thus better wash and ream. Various additives can be used with water-base muds as well – to reduce cost and environmental problems of OBM - which can improve borehole stability and thus help in obtaining high quality wellbore^91,92^.

#### Model-3: tripping and casing running speeds prediction

The third model estimates tripping and casing running speeds, key components of overall section time. A high-quality hole will experience a smoother friction profile and thus better tripping and casing running speeds.

- Directional technology is the main factor to be addressed in terms of hole condition as RSS is known for its superiority over PDM in terms of hole quality.
- Formation also determines how good a trip or casing running would be. Troublesome and unstable formations will suffer longer tripping times. Formations differ among different fields, and a troublesome formation at certain field may be a no-risk formation at another.
- The speed with which the hole was drilled is known between drilling professionals to have a major effect on how good a trip will be. A trade-off between ROP and tripping speed is sometimes necessary.
- Bit type also affects tripping speed in different aspects. Tri-cone bits usually are easier to run compared to Polycrystalline diamond compact (PDC) ones, where a PDC bit with back-reaming cutters may be easier to pull.
- Inclination and DLS also have a dominant effect on tripping speed. High inclination and DLS are associated with higher T&D and thus slower tripping and casing running on average.
- The type and direction of the trip affect how fast it is. Liner running is usually faster than casing running, also running speeds are usually higher than pulling speeds.

In addition, the second trip-out of hole is usually better than the fast one if time-dependent wellbore instability is absent.

#### LIH probability

LIH probability is usually higher in case of PDM than RSS due to obvious reasons. The expected cost value of such an event can be estimated as PLIH multiplied by CLIH (where; PLIH is the probability of losing tool in hole and CLIH is the tool cost of lost in hole ($)) and added to the total cost if required. The authors recommend using a suitable value based on area experience.

Table 2 shows a summary for feature engineering of the three models grouped by data category.

While most data in Table 1 are straightforward, more elaboration may be required for the last one, i.e., tripping type and direction. A hole in a good shape would allow better tripping profile, either while tripping in or out and either while tripping drill string or running casing/liners. The tripping type (drill string, casing, or liner) and direction (in or out) are used amongst other variables to predict the tripping speed which is used as the hole quality quantifier. Tripping speed is never constant and for the sake of simplicity an averaged value for different intervals is used in this model.

**Table 2:** Summary of Feature Engineering for the three machine learning models.

	Model number →	Model-1	Model-2	Model-3
	Model output →	Bit ROP	Average ROP	Tripping/Casing running speed
Input data category ↓	Feature used ↓			
Directional survey	MD	√		
	Inclination	√	√	√
	DLS	√	√	√
Drilling equipment & tools	Rig		√	√
	DD_Tech	√	√	√
Lithology	Field	√		√
	Formation	√		√
Hole configurations	Hole size	√	√	√
Drilling parameters	WOB	√		
	Bit RPM	√		
	Flow rate	√		
	Bit ROP		√	√
Mud records	Mud type	√	√	√
	Mud weight	√		
Bit record	Bit type	√	√	√
	Bit condition	√		
Tripping data	Tripping type/Direction			√

### Workflow

In this section, we outline the systematic approach undertaken to develop our machine learning models for predicting drilling performance metrics. The workflow begins with data acquisition, where historical field data is collected to ensure a robust foundation for analysis. Following this, we detail the data pre-processing steps, which involve refining the dataset by addressing missing values and normalizing features to enhance model performance. Finally, we discuss the optimization of model hyperparameters, a crucial phase that fine-tunes the algorithm to accurately capture underlying patterns while minimizing overfitting. This structured methodology underpins the effectiveness of our predictive models and their applicability in real-world drilling operations.

#### Data acquisition

A comprehensive dataset was collected from wells drilled in three distinct fields between 2018 and 2020, employing both PDM and RSS technologies. The data encompassed a wide range of input features essential for training the machine learning models, including drilling parameters, lithology descriptions, mud and bit records, directional surveys, and drilling reports.

**Fig. 3:**
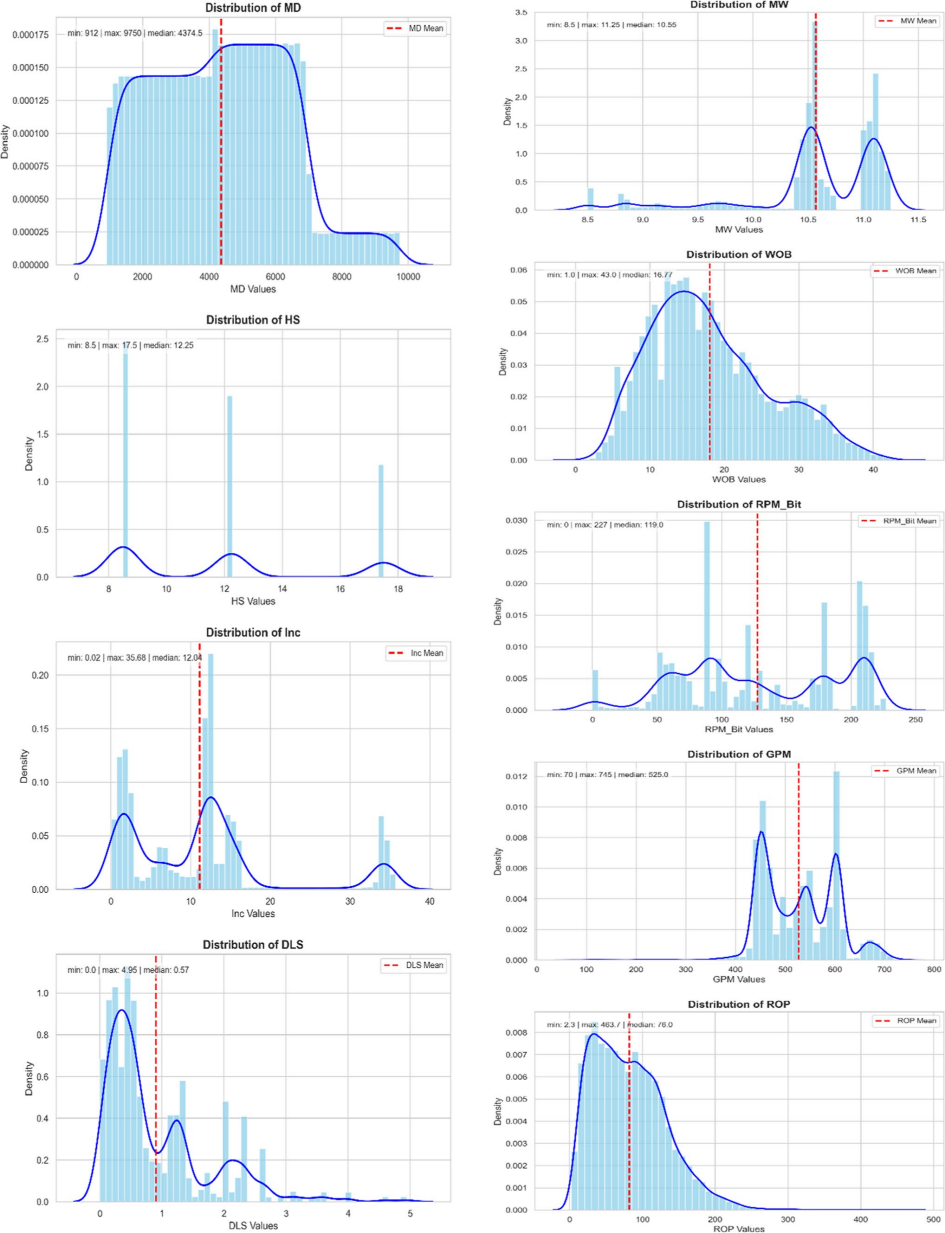
Histogram of numerical data distribution used in model-1.

**Fig. 4:**
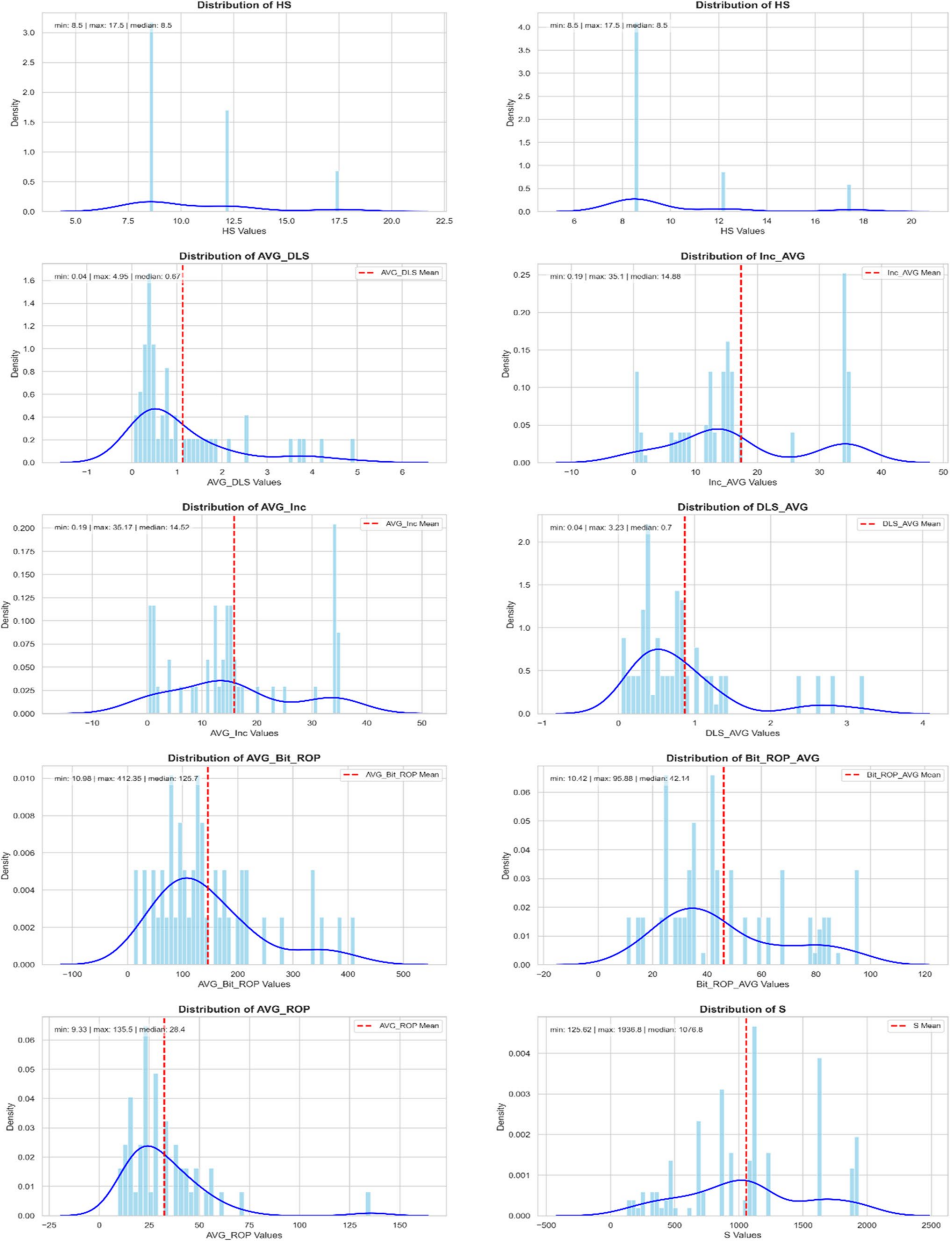
Histogram of numerical data distribution used in model-2 (to the left) and model-3 (to the right).

The initial dataset comprised 17 distinct feature categories, with a total of 10,691 data points per category, amounting to 181,747 data entries overall. Figures 3 and 4 illustrate the distribution histograms of the numeric input features utilized for each model.

#### Data pre-processing

Before delving into the specifics of data processing and manipulation, it is important to note that the subsequent computational tasks were carried out using Pandas^93^ (a Python^94^ library) for data handling, the Python programming language for implementation, and the Scikit-learn library^95^ for machine learning functionalities. Additionally, eXtreme Gradient Boosting (XGBoost)^96^ was employed as the machine learning algorithm for this study. These tools provide a robust framework for efficiently managing data and applying various preprocessing techniques, with Scikit-learn facilitating feature scaling and model evaluation, while XGBoost serves as a powerful algorithm for model training, ensuring a solid foundation for the development of the machine learning models.

The data preprocessing phase involved carefully excluding intervals where drilling was conducted without the use of either RSS or PDM. Additionally, we eliminated data points exhibiting illogical values, such as negative rates of penetration (ROP < 0) or weight on bit (WOB < 0). As a result, the final dataset consisted of 8,366 rows and 17 columns of validated data, ensuring a high-quality foundation for our analysis.

Data was split between training (80%) and testing (20%) sets using (Stratified Shuffle Split) to maintain the distribution of features between both sets. Missing values were handled using median imputation for numerical features and most-frequent-value imputation for categorical features. Numerical features were then standardized using Standard Scaler technique to ensure uniform contribution across all features. For categorical variables, (One-Hot Encoding) was applied to convert categorical values into binary vectors, preventing any ordinal assumptions. No manual feature selection was performed, as all variables were deemed relevant based on domain knowledge. A custom-built transformation pipeline was employed to automate the entire process, using Scikit-learn ColumnTransformer function to handle both numerical and categorical data simultaneously. This allowed preserving the integrity of the data while making it suitable for machine learning tasks. The final output was an array of fully prepared data, ready for model training and evaluation.

#### Model parameters optimization^96^

For hyperparameter optimization, we employed GridSearchCV with 5-fold cross-validation to fine-tune the XGBoost model. In 5-fold cross-validation, the dataset is split into five equal parts, or “folds.” The model is trained on four of these folds while the remaining fold is used for testing. This process is repeated five times, with each fold serving as the test set once. The final performance is averaged across all five runs, which ensures that the model is not biased toward any particular subset and provides a more reliable estimate of its performance on unseen data. This method helps assess the model’s generalization ability and reduces the risk of overfitting.

GridSearchCV allows us to identify the best combination of hyperparameters that optimize the model’s accuracy. Specifically, we fine-tuned parameters such as the learning rate (which determines how fast the model learns), the maximum depth of trees (which controls model complexity), the number of estimators (how many trees are built), and the subsample and colsample_bytree (which determine the proportion of data and features used in each iteration). Through careful tuning, we ensured that the model captures essential patterns while minimizing overfitting, ultimately enhancing its accuracy and reliability.

## Results and discussion

Various machine learning models perform differently depending on the problem at hand, and the most reliable way to assess a model’s effectiveness is through rigorous training and testing on respective datasets. In this study, six models were evaluated: linear regression, decision tree, random forest, support vector machine (SVM), stochastic gradient descent (SGD), and XGBoost. Among these, XGBoost demonstrated the highest accuracy and the least susceptibility to overfitting, indicating its superior ability to generalize to new datasets without compromising performance.

In addition, XGBoost was chosen due to its ability to handle complex, high-dimensional data from diverse sources such as drilling parameters, lithology, and operational records. Its built-in regularization prevents any single feature from dominating the model, ensuring unbiased and reliable predictions. XGBoost natively handles missing data, minimizing the need for extensive preprocessing. Additionally, its support for hyperparameter tuning and gradient boosting optimizes model performance. Given the potential to integrate more fields and operational settings, XGBoost’s support for parallel and distributed computing will allow the model to scale efficiently as more data becomes available, making it an ideal choice for diverse drilling environments.

### Performance metrics of the models

**Fig. 5:**
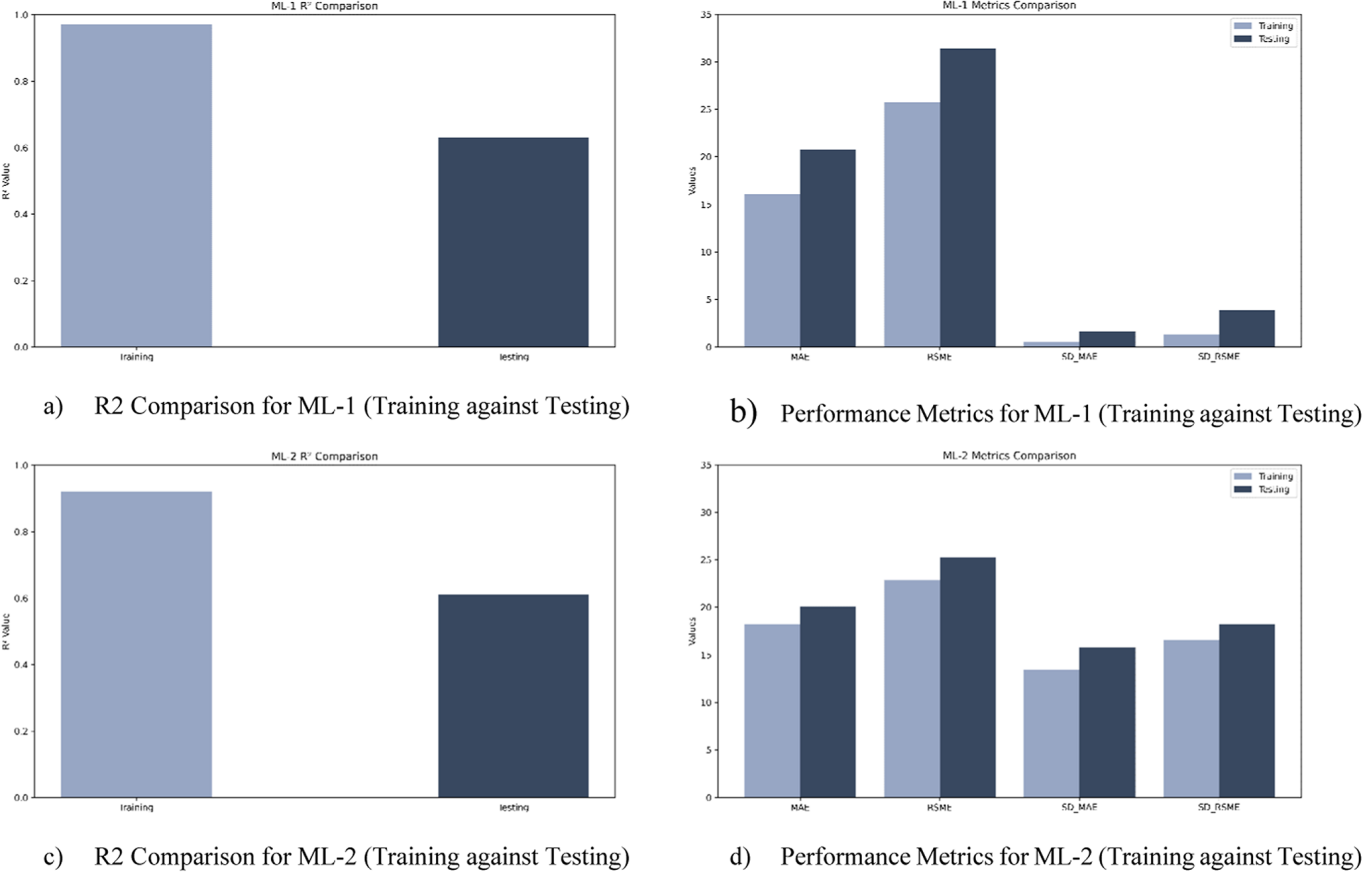
Performance Metrics and R2 comparison (Training against Testing) for ML-1 and ML-2.

**Fig. 6:**
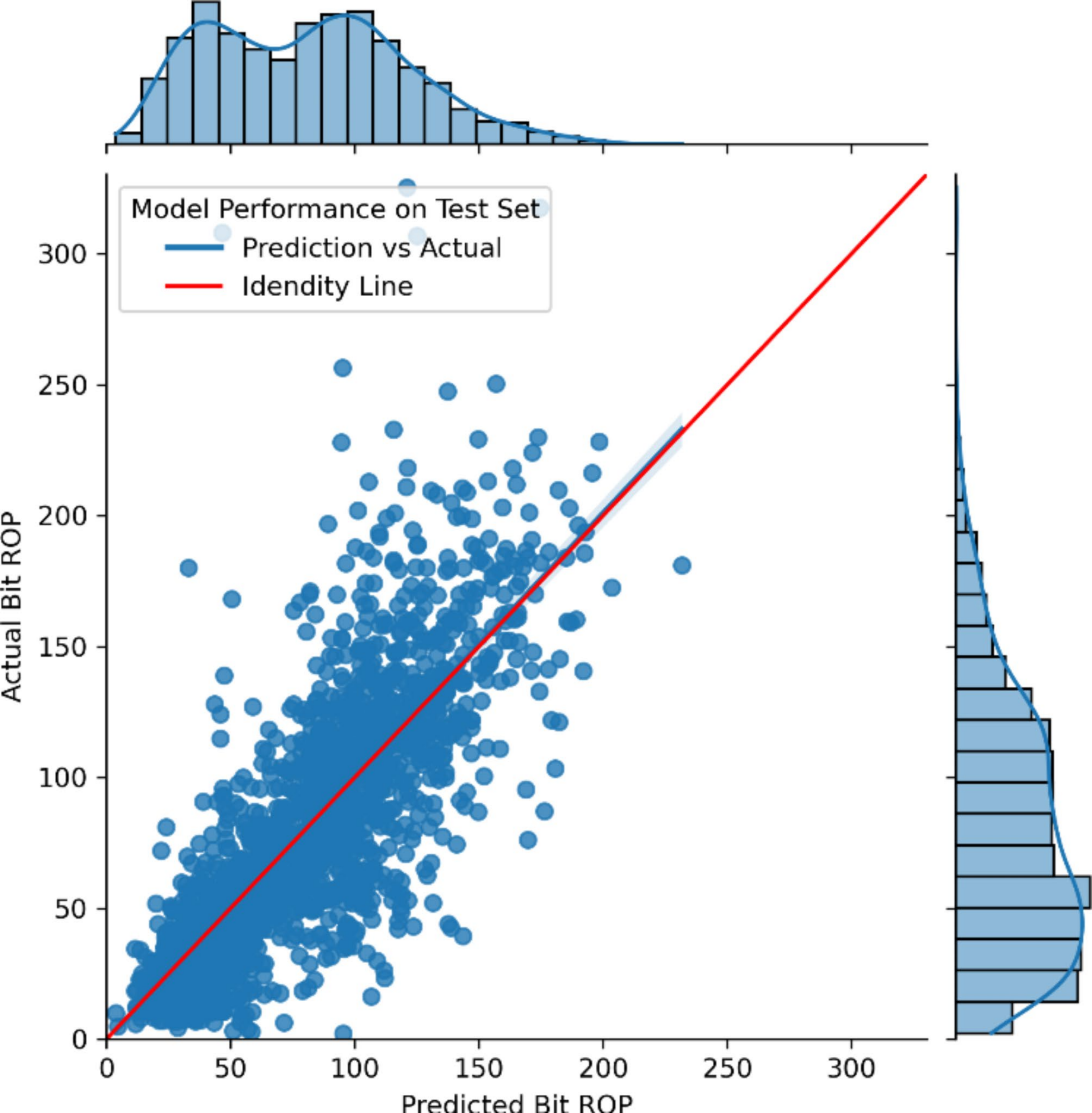
Performance of Model-1.

**Fig. 7:**
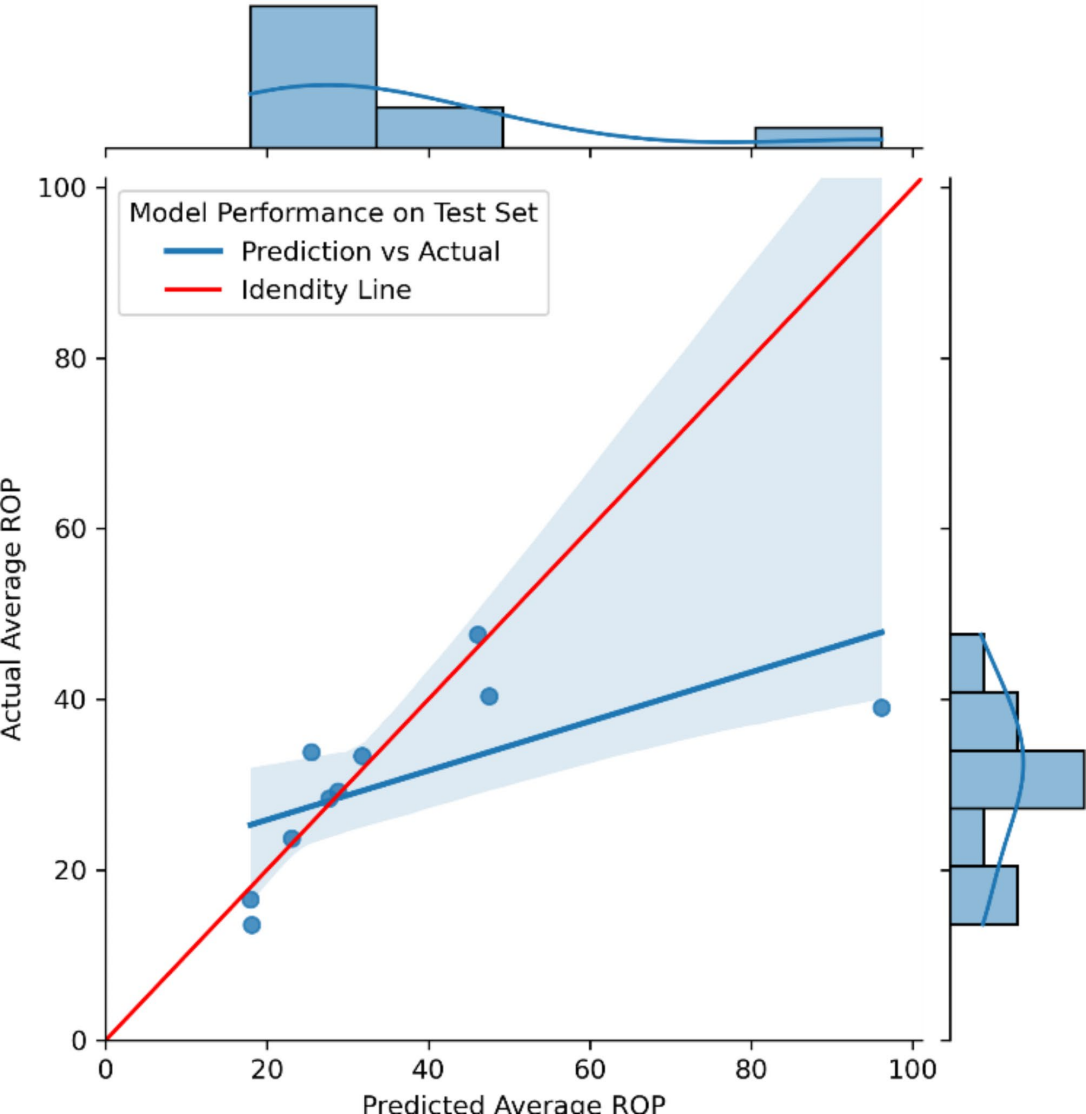
Performance of Model-2 (prediction of average ROP).

Model-1 demonstrates strong predictive capability for Bit ROP, achieving R-squared values of 0.97 and 0.63 for training and testing datasets, respectively, with Mean Absolute Errors (MAE) of 16.06 and 20.76 ft/hr, indicating minimal overfitting. Model-2, which utilizes predictions from Model-1 along with required features to estimate Average ROP, shows generalizability with MAEs of 18.24 and 20.12 ft/hr on training and testing datasets, respectively. Model-3 effectively predicts tripping speeds with an average MAE of 83.70 ft/hr (less than one stand per hr error) and a standard deviation of 22.96 ft/hr, though it has not been validated with independent testing data due to dataset limitations. The performance metrics for ML-1 and ML-2 are illustrated in Fig. 5, and the parity charts for both models are illustrated in Figs. 6 and 7.

### Application of the model and responsiveness to input variation

After validating the model, it was applied to three independent planned sections with diverse geological setting and well configurations to check model response to different inputs. Output results obtained from the model were plugged in Eq. 1 through 3 to estimate total section time and cost using both PDM and RSS.

**Fig. 8:**
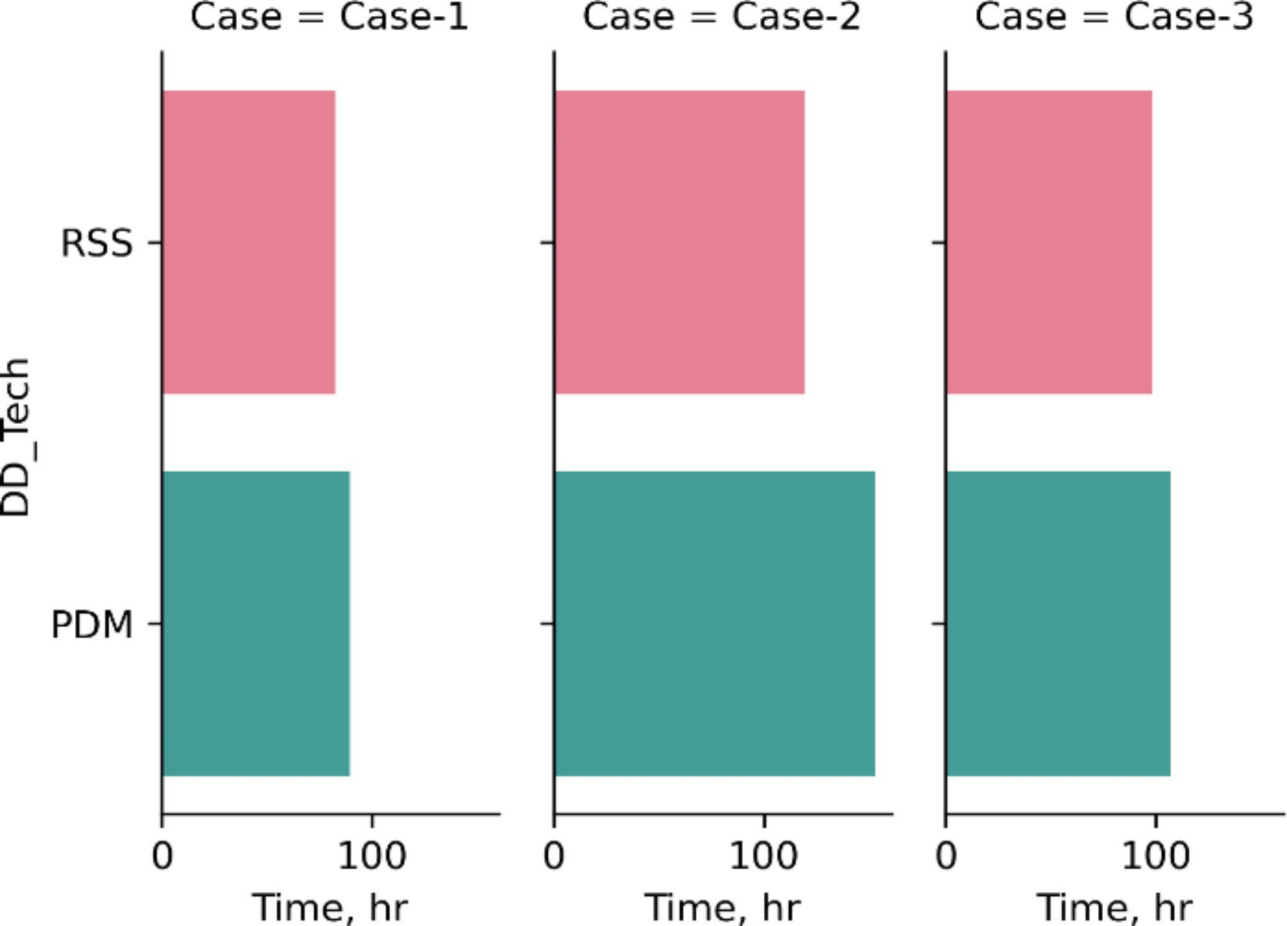
Results of section time for three different cases interpreted using the model.

**Fig. 9:**
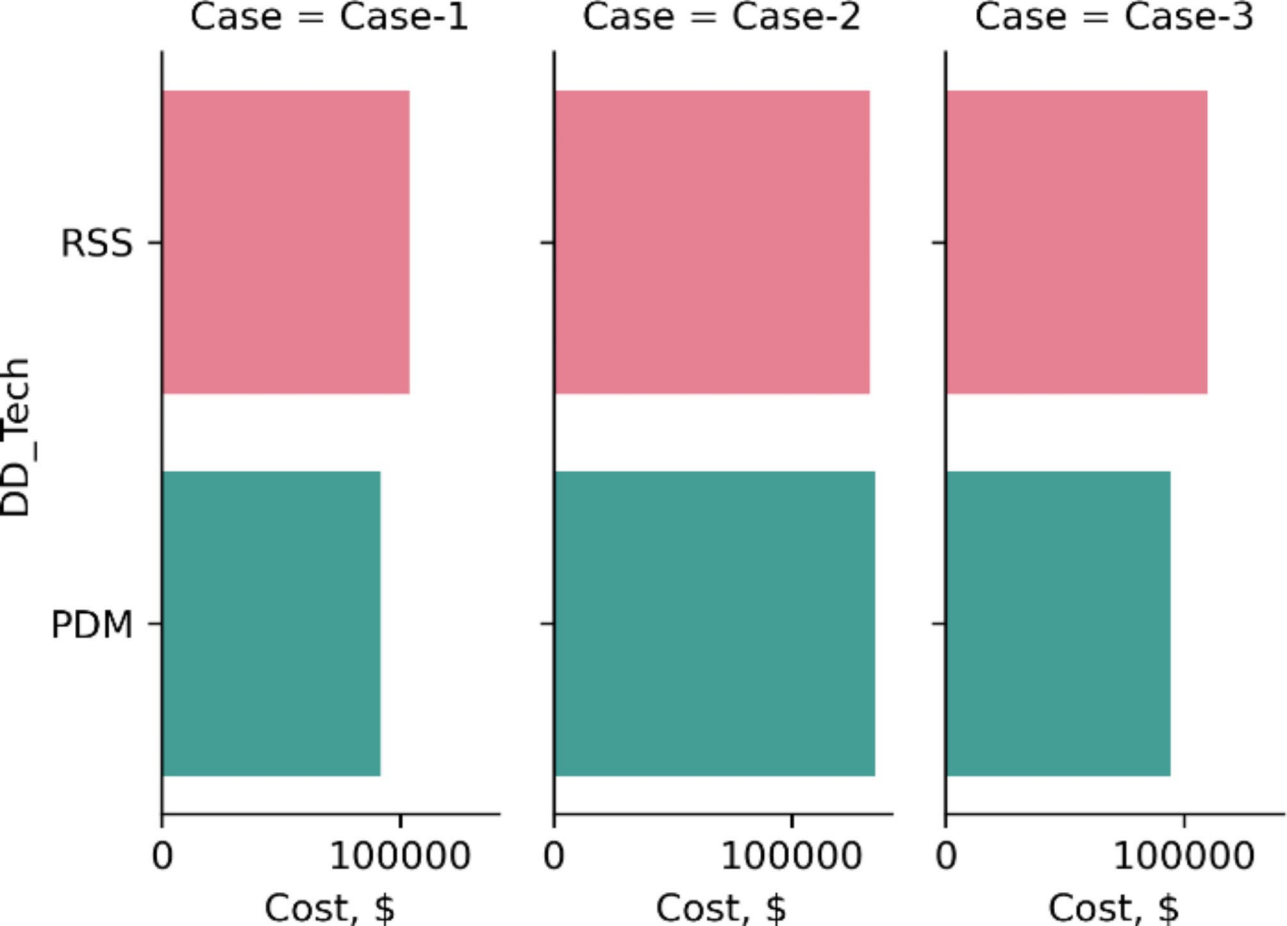
Results of section cost for the same cases interpreted using the model.

Figures 8 and 9 show the result obtained using the model for three different cases with entirely different input features, which clearly assures the sensitivity of model output depending on circumstances of a certain drilling operation. For example, in case-2, the operator must drill the section using RSS, as it is almost the same price (2,000 $ less as per case cost scheme) and would finish the Sect. 33 h earlier than PDM. Where in cases 1 & 3, RSS section cost is projected to be 12,000$ and 15,000$ more expensive and expected to finish the Sect. 7 h and 9 h earlier than PDM respectively. And here the operator would decide the tool based on his custom requirements.

### Insights from results of model application

**Fig. 10:**
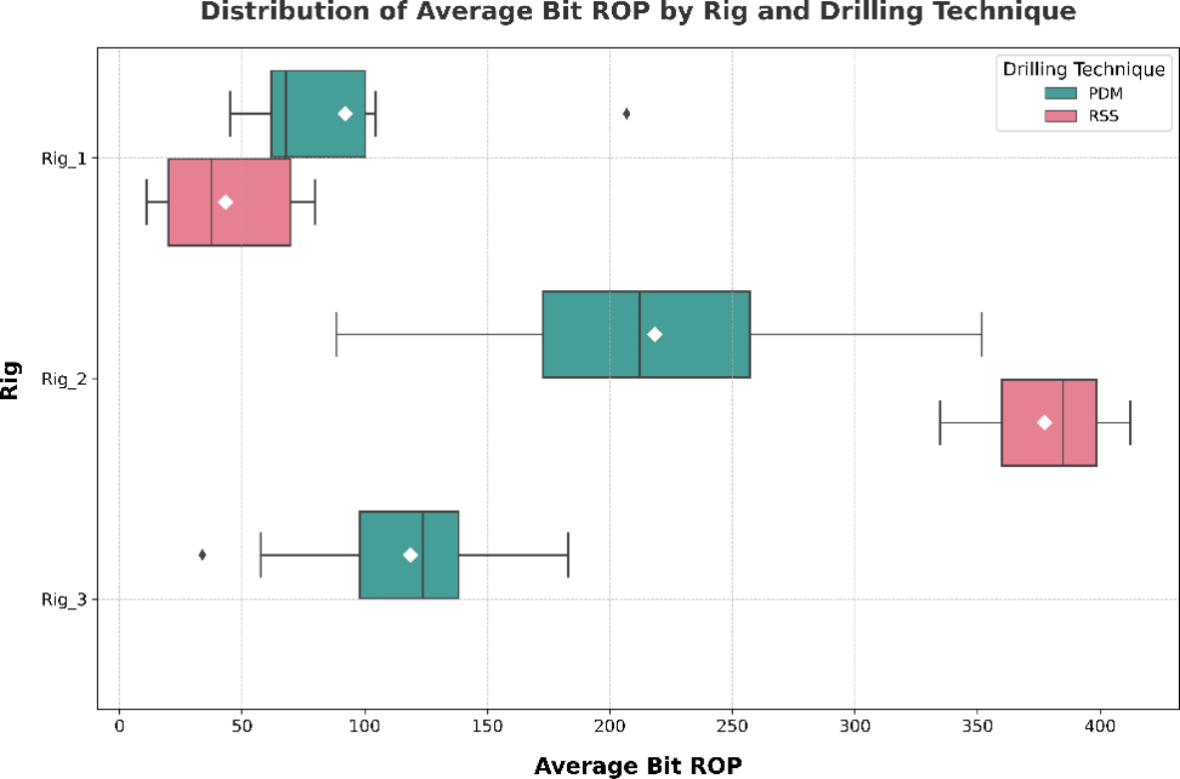
The effect of transforming rigs with small hydraulic HP to RSS.

*Effect of rig equipment*: One important result was that the model is sensitive to the rig used. Some rigs made astonishing results by converting to RSS both theoretical and actual. The big performance leap with about 100% increase in Bit ROP (in case of Rig_2, see Fig. 10), can be attributed to low HP mud pumps, which limits WOB that can be used PDM (higher WOB should be accompanied with higher flow rate to avoid stalling). After using RSS, the performance showed a great improvement.

**Fig. 11:**
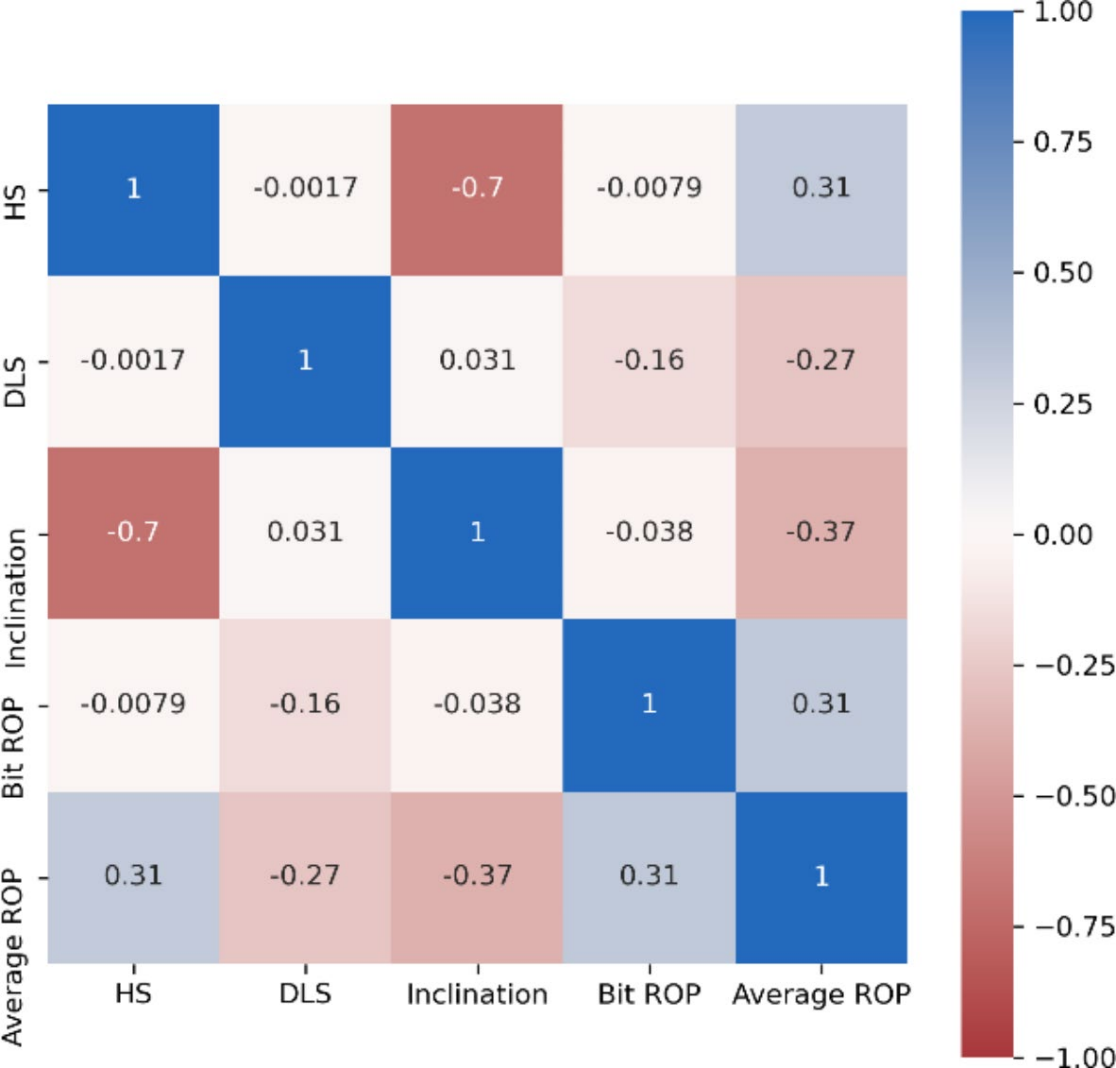
Pearson correlation heat map between various features of Model-2.

*Criticality of domain knowledge for data analysis*: Domain knowledge is of utmost importance when dealing with data-driven models. One observation was that a positive correlation exists between hole size and Bit ROP (Fig. 11), the noticed correlation is not a result of cause/effect relationship, but an intuitive result of the fact that bigger diameter sections are shallower, hence the actual correlation is a negative correlation with Measured Depth (MD), not a positive one with hole size.

**Fig. 12:**
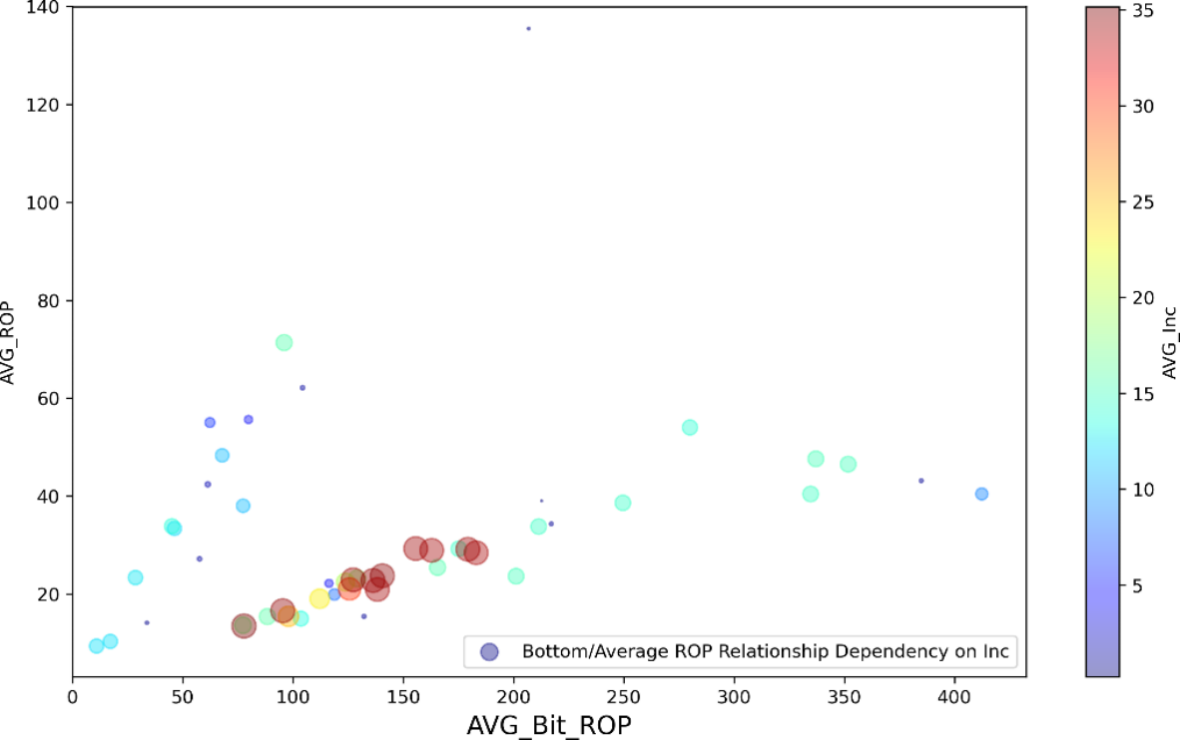
Relationship between Bit ROP and average ROP.

*On-bottom/average ROP relationship dependency on inclination*: Although the relationship between Bit ROP and Average ROP is a direct linear relationship, the linearity coefficient changes with inclination. At low inclinations, Average ROP is very sensitive to Bit ROP, where it is less sensitive at higher inclination, which can be attributed to higher T&D at high inclination which require more carefulness during wash and ream and hence less average ROP (Fig. 12).

**Fig. 13:**
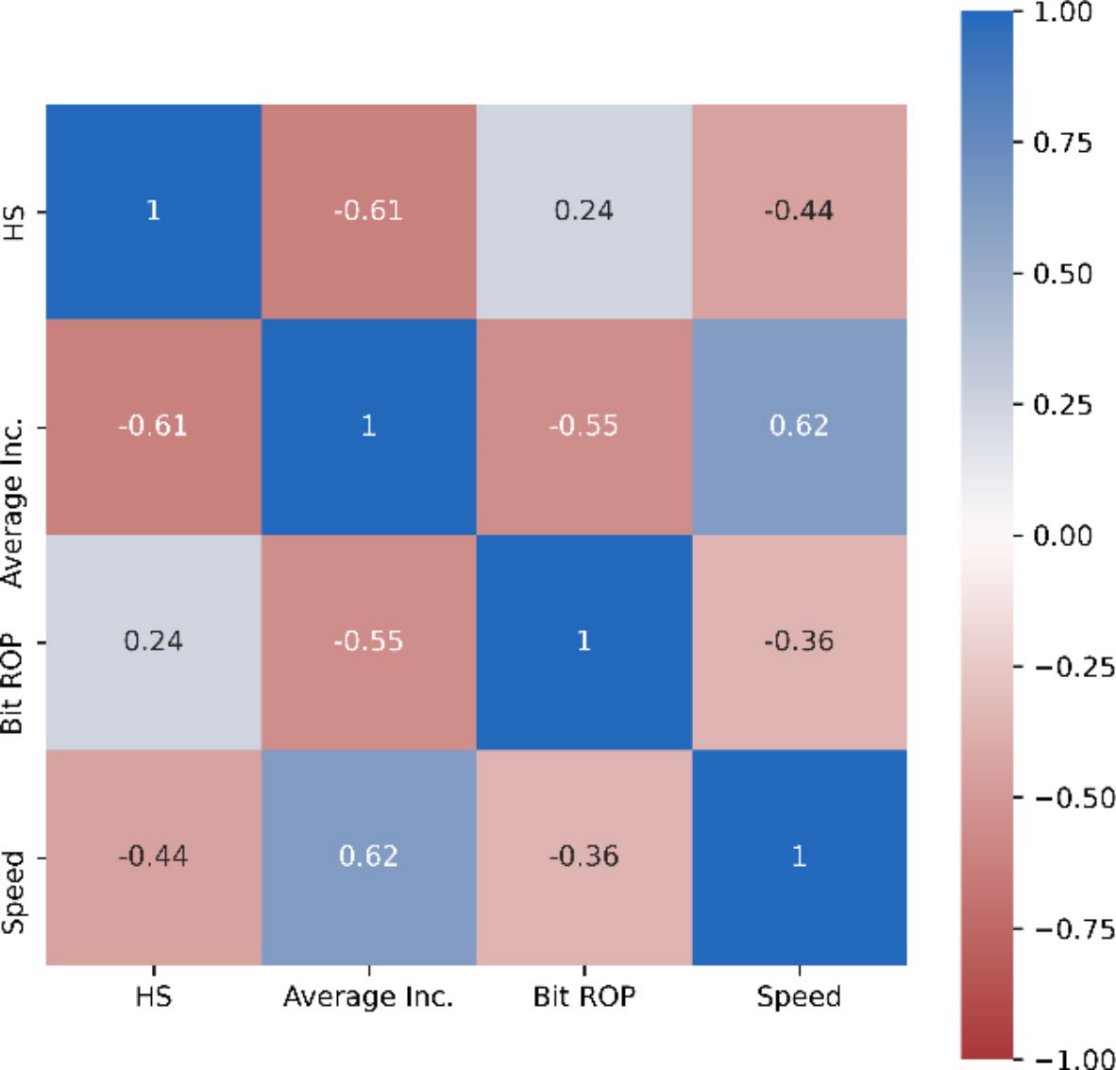
Inclination and Bit ROP Pearson correlation with tripping speed.

*ROP/tripping speed relationship*: Model-3 provides two interesting results, that there is a negative correlation between average ROP and tripping speed, which supports the current knowledge of adverse effect of fast drilling on tripping quality (Fig. 13).

One other interesting result is the positive correlation between hole inclination and tripping speed, which may seem counterintuitive, but this is mostly attributed due to this is linked with the fact that highest angles occur in the hold section within the study wells, which is usually easier for tripping, as more wells with different directional design are added to the model, it will correct itself.

The main take-away of the paper is that neither PDM, nor RSS is the optimum steering tool in every situation. The optimum technology changes on a well-by-well basis, and the aim of this paper is to present a data-driven model that uses past performance figures to automate the selection steps and makes it a timely and cost-effective process.

## Conclusions and recommendations

This study developed and implemented a series of machine learning models to optimize the selection of directional drilling technologies—specifically, Positive Displacement Motors (PDM) and Rotary Steerable Systems (RSS)—under varying operational conditions. Based on the results, the following conclusions can be drawn:

1. By leveraging historical data, the models accurately estimate total section time (including drilling and tripping phases) and associated costs based on specific inputs.
2. The developed models offer a rapid, reliable, and robust decision-making tool for operators, facilitating informed choices about the most efficient directional drilling technology in terms of cost and time.
3. The developed model proved to be responsive to change of input data and give clear and sharp recommendations.
4. Recommendations derived from model applications indicate that RSS technology demonstrates a favorable economic application on smaller rigs equipped with compact mud pumps.
5. The study emphasizes that well-specific conditions are critical determinants in selecting the optimal directional tool; no single tool universally excels across all scenarios.
6. The analysis identified that the relationship between on-bottom ROP and average ROP is inclination-dependent.
7. Domain knowledge and understanding were crucial in selecting relevant input features and interpreting model outputs, ensuring the machine learning model provided actionable and accurate recommendations for field operations.

The findings of this study indicate that incorporating an additional step in the planning phase for each directional well is essential for selecting the appropriate drilling tool, particularly in situations where no single technology emerges as the definitive choice. Operators often favor Positive Displacement Motors (PDM) for economically straightforward wells, while Rotary Steerable Systems (RSS) are preferred for more challenging conditions. Our model effectively identifies the optimal tool for a significant majority of contemporary wells, enhancing decision-making related to drilling time and cost efficiency.

However, it is important to acknowledge certain limitations that we consider as Prospects for future research directions.

1. Enhancing Data Quality for Future Insights: The dataset utilized for machine learning model-3 has a relatively small number of data points, particularly for tripping and casing running speeds. Instead of having data recorded every foot, we have average speeds over certain intervals, which may limit the model’s precision in predicting tripping speed. As more wells are drilled and data accumulates, the accuracy of this model is expected to improve.
2. Expanding Tool Availability for Improved Applications: The current models are based solely on data for PDM and RSS. The inclusion of data from motorized rotary steerable systems (MRSS) could enhance the model’s comprehensiveness. However, due to the limited use of MRSS in the field, we currently lack the necessary data to include this tool.
3. Advancing Feature Selection for Enhanced Model Performance: One additional feature that could be beneficial is the date when each well was drilled. This would allow the model to account for learning curve effects on performance over time. However, this study focused on wells drilled within a narrow time window, limiting the applicability of this feature.

Looking ahead, the work presented here advocates for a paradigm shift in how directional drilling tools are selected, emphasizing the importance of data-driven methodologies to optimize performance across diverse drilling environments. As the industry continues to evolve, the integration of advanced machine learning techniques will undoubtedly play a pivotal role in shaping the future of drilling operations. Additionally, exploring the incorporation of other diagnostic tools could provide a more holistic approach to optimizing drilling operations. By addressing these avenues, the potential for significant advancements in drilling efficiency and cost-effectiveness can be realized, ultimately benefiting oil and gas operators.

## Data Availability

The raw data supporting the conclusions of this article will be made available by the authors on request (contact: Muhammad Nour muhammadnour@icloud.com).

## Abbreviations

BHA: Bottom hole assembly
BUR: Build up rate
DLS: Dog leg severity
HP: Horsepower
HP-PDM: high performance PDM
LIH: Lost in hole
MAE: Mean absolute error
ML: Machine learning
PDC: Polycrystalline diamond compact bit
PDM: Positive displacement mud motor
RMS: Root mean square
ROP: Rate of penetration
RPM: Revolution per minute
RSS: Rotary steerable system
T&D: Torque and drag
TDS: Top drive system
WOB: Weight on bit
XGBoost: eXtreme Gradient Boosting

## Nomenclature

C: Total cost ($)
C_f_: Flat charge of either PDM or RSS
C_r_: Rig operating rate including service companies ($/hr)
C_t_: Tool operating rate (PDM or RSS), including all related BHA components ($/hr)
F: Footage drilled (ft)
N: Number of trips
ROP_AVG_: Average run ROP including tool face adjusting, downlinks, surveys, connections, wash, and ream time (ft/hr)
S_c_: Casing or liner running speed (ft/hr)
S_PC_: Tripping speed while pulling within cased hole (ft/hr)
S_PO_: Tripping speed while running within open hole (ft/hr)
S_RC_: Tripping speed while running within cased hole (ft/hr)
S_RO_: Tripping speed while running within open hole (ft/hr)
T: Total time (hr)
TD: Section total depth (ft)
T_MU_: Make Up time of Tool (hr)
T_LD_: Time of lay down (hr)
T_t_: Tripping time (hr)
